# Spinal cord injury as an indicator of abuse in forensic assessment of abusive head trauma (AHT)

**DOI:** 10.1007/s00414-021-02526-x

**Published:** 2021-02-22

**Authors:** Michela Colombari, Claire Troakes, Stefania Turrina, Franco Tagliaro, Domenico De Leo, Safa Al-Sarraj

**Affiliations:** 1 Department of Diagnostics and Public Health, Section of Forensic Medicine, University of Verona, Verona, Italy; 2 Institute of Psychiatry, Psychology & Neuroscience, King’s College London, London, UK; 3 Institute of Translational Medicine and Biotechnology, Sechenov First Moscow State Medical University, Moscow, Russia; 4 Department of Clinical Neuropathology, King’s College Hospital NHS Foundation Trust, London, UK

**Keywords:** Abusive head trauma, Spinal cord injury, Spinal subdural haemorrhage, Shaken baby syndrome, Forensic investigation

## Abstract

Abusive head trauma (AHT) in children is notoriously one of the most challenging diagnoses for the forensic pathologist. The pathological “triad”, a combination of intracranial subdural haematoma, cerebral oedema with hypoxic-ischaemic changes and retinal haemorrhages, is frequently argued to be insufficient to support a corroborated verdict of abuse. Data from all available English-language scientific literature involving radiological and neuropathological spinal cord examination is reviewed here in order to assess the contribution of spinal cord changes in differentiating abusive from accidental head trauma. In agreement with the statistically proven association between spinal subdural haemorrhage (SDH) and abuse (Choudhary et al. in Radiology 262:216–223, 2012), spinal blood collection proved to be the most indicative finding related to abusive aetiology. The incidence of spinal blood collection is as much as 44–48% when all the spinal cord levels are analysed as opposed to just 0–18% when the assessment is performed at cervical level only, in agreement with the evidence of the most frequent spinal SDH location at thoracolumbar rather than cervical level. In this review, the source of spinal cord blood collection and how the age of the child relates to the position of spinal cord lesions is also discussed. We concluded that the ante mortem MRI examination and post mortem examination of whole-length spinal cord is of fundamental interest for the assessment of abuse in the forensic setting.

## Introduction

Abusive head trauma (AHT) in children including “Shaken Baby Syndrome” refers to intracranial traumatic brain injury in child victims of abuse. Shaking (with or without impact) has been identified as the leading mechanism resulting in the common AHT features, namely, the “triad” of intracranial subdural haematoma, cerebral oedema with hypoxic-ischaemic changes and retinal haemorrhages [1]. The term shaken baby syndrome (SBS) has been used for decades, since Caffey introduced the term “whiplash shaken infant syndrome” in 1974 to describe a combination of intracranial and extracranial findings in abuse, in the absence of external signs of violence [2]. AHT is a well-known cause of mortality, morbidity and disability, usually but not exclusively, in children under 1 year of age. Infants have high weight heads and weak neck muscles, and when a very young baby is shaken, the head moves repeatedly and excessively forwards and backwards causing excessive acceleration and deceleration of the brain. The precise mechanism and pathophysiology are usually challenging to determine, although many injuries are caused by violent hyperflexion and hyperextension, resulting in a condition similar to that now called shaken baby syndrome (SBS), but others are caused by blunt trauma (impact) as well as by the combination of the two mechanisms. The incidence of AHT has been reported to be as high as 15–29 per 100,000 infants but is probably underestimated [3]. The pathological features in the brain have been described in many articles as a constellation of pathological features, centred on demonstration of the triad of subdual haematoma and hypoxic encephalopathy together with retinal haemorrhages [4]. However, these have been determined to not be sufficient to confirm abuse by a systematic review from the Swedish Agency for Health and Technology Assessment [5]. Several efforts to find additional pathological features associated with AHT have focused mainly on cervical spinal cord, due to the mechanism of shaking and the violent head and neck movements. Although pathological changes at this level have been reported, to date, they are not firmly embedded with the triad as solid diagnostic criteria. The present article is a review of the current scientific knowledge regarding spinal cord injuries in children suffering from AHT in order to better understand the role of spinal cord examination in forensic assessment of child abuse.

### Intracranial AHT pathological findings

Since Guthkelck suggested the leading role of shaking in the development of brain subdural collections usually observed in battered babies in 1971, the detection of intracranial subdural haematoma (SDH) has become the cardinal point for the diagnosis of shaken baby syndrome [6]. When Caffey (1974) coined the term “whiplash shaken infant syndrome” for the first time, the common clinical manifestation consisted of SDH in association with extracranial findings such as intraocular bleeding [2]. The observation was subsequently confirmed by a wide range of studies on AHT children [7–13]. The association of extra-axial bleeding with inflicted head injuries was statistically proven by Dashti et al. (1999) who studied 32 AHT children under the age of 2 years, in comparison with a group of 68 accidentally injured babies [14]. In this study, the SDH appeared drastically more frequent in the AHT cohort (69% vs 7%, *p* < 0.001). The result was confirmed by Vinchon et al. (2010) who reported the percentage of children with SDH to be as high as 82.2% in those with AHT compared with only 43.6% in the control group (*p* < 0.001) [15]. Moreover, in a comprehensive systematic review by Kandom et al. (2014), the presence of subdural collections on neuroimaging was related to abuse by an odds ratio of 8.2 (95% CI) [16]. Intracranial subdual haematoma is usually observed at imaging examination as a small amount of SDH with compressed cerebral sulci, displaced corticodural veins, sometimes in association with subdural membranes [17]. Despite the initial description of SDH in AHT as chronic, the most common observation of SDH has been that of blood collection of recent onset [18, 19], and a review of all the English-language literature on shaken baby syndrome over a 32-year period demonstrated the higher prevalence of acute blood collections rather than chronic [20]. In agreement with the description that “interhemispheric haemorrhages and spinal SDH in multiple sites or of different densities were almost exclusively seen in AHT” [21], intracranial SDH collection is nowadays well known to have a different densities aspect, and the association of abusive head trauma with mixed intensities SDH on CT scan has been statistically proven (*p* < 0.001) [19]. Furthermore, paediatric patients with SDH of different intensities were found to be more likely to suffer from abusive head trauma (OR 6.39, 95% CI) [22]. The distribution of SDH has been more substantially assessed, and it usually appears as a unilateral or bilateral thin film of subdural collection over the convexities, with a particular predisposition for the interhemispheric fissure [23]. Adamsbaum et al. (2010) reported SDH incidence up to 95.5% in the interhemispheric fissure, 86% in the tentorium cerebelli and 100% in the right or left lateral spaces [24]. The predominantly supratentorial locations have been proposed to be the hallmark of AHT and the main tool to distinguish AHT-associated haematomas from those related to birth [25]. In a study where the intracranial imaging result was compared with the mechanism of trauma, 73% of interhemispheric SDH resulted from intentional injuries as well as 72% of SDH over the convexity [26]. The significant association between the interhemispheric location of blood collections and AHT has been also statistically proven (OR 9.5, 95% CI) [21]. Surprisingly, when Barlow et al. (1999) studied 12 children admitted to hospital with a diagnosis of abusive head injury on MRI, the most common site of SDH appeared to be the subtemporal area, but the result should be read with the limitation of the weakness of CT to investigate this area and thus the possibility of overlooking them in many cases [27]. Subdural collections in the brain of AHT children has been confirmed at post mortem examination [27–31]. The results from 53 AHT autopsied cases allowed Geddes et al. to recognize an age-related pattern of injuries [32]. Infants less than 1 year old usually presented with a bilateral, thin film of SDH as opposed to older children who showed large and localized subdural haematomas. The older group presented frequently with axonal damage in the hemispheric matter and with extracranial injuries. The younger group, on the other hand, was more prone to the presence of axonal damage at the craniocervical junction and to skull fractures. Subdural collection usually occurs in association with other intracranial findings, such as subarachnoid haemorrhage and cerebral oedema.

Cerebral oedema and hypoxic-ischaemic changes are features commonly associated with abuse [12, 33, 34]. Wells et al. (2002) and Keenan et al. (2004) demonstrated that cerebral oedema is more common in abused babies than in those accidentally injured (78% vs 13% and 31% vs 13%) [18, 26]. The relationship between abuse and cerebral oedema has been statistically proven (*p* < 0.002) (OR 2.2, 95% CI) as well as that between abuse and hypoxic-ischaemic injuries (OR 3.7, 95% CI) [15, 35]. Subarachnoid haemorrhage (SAH) is frequently reported in AHT children with a higher incidence rate in autopsied children (50% Geddes et al. 2001, 92% Brennan et al. 2009) than those investigated through imaging (18% Dashti et al. 1999 on CT and MRI) [14, 32, 36]. When AHT children are compared with accidentally injured babies, SAH is seen more frequently in the accidental group (33% vs 61% respectively in Wells et al.’s study (2002) and 11.3% vs 22.7% in Keenan et al.’s study (2004) [18, 26].

Intraparenchymal changes are sometimes reported in association with the above-discussed intracranial injuries. Although Brennan et al. (2009) found a peculiarly high incidence of them between AHT babies (66% AHT had intracerebral bleeding, 65% had superficial cerebral contusion and lacerations, 65% had deep cerebral contusion and lacerations), intraparenchymal injuries are usually referred to as a sporadic finding [36–38].

Skull fractures in young babies are seen more commonly in association with extradural haematoma (EDH) which is, in its turn, an uncommon finding in abused babies [21]. Children suffering from abuse presented less frequently with skull fractures than those accidentally injured (57% vs 30.4%) as opposed to multiple skull fractures which are more frequently observed among those abused (14.2% vs 2.7%) [25].

### Spinal cord in AHT: evidence from neuroradiology

#### Spinal blood collections

Evidence of spinal cord involvement in suspect of abuse comes mostly from radiological investigation performed at the moment of hospital admission when brain and spinal cord are imaged through CT scan or MRI. In 1994, Diamond et al. published a case report of a 12-month-old female admitted to the hospital with T12 over L1 anterior spondylolisthesis [39]. The MRI scan showed a T12-L3 pre-spinal mass possibly of haemorrhagic nature and tethered cord. A court confirmed the diagnosis of child abuse, but no further information on the mechanism of trauma was given. Three years later, Feldman et al. (1997) analysed 12 AHT children at cervical level in an attempt to assess the convenience of MRI in detecting the AHT cases [40] (Table 1). As opposed to the positive results from post mortem examinations in five deceased children which successfully managed to detect spinal blood collections (subdural haematoma on the upper cervical cord in one child along with subarachnoid collections between the remaining three children), the MRI failed to detect any signs of blood collections. The little sensibility of the MRI methodology at the time the study was performed could possibly explain the poor data. When Koumellis et al. (2009) analysed the spinal MRI findings between 18 AHT children (mean age 3 months) admitted to a tertiary neuroscience centre over a 7-year period in 2009, they showed completely different outcomes [50]. The examination was performed on the whole spinal column, and almost half (44%) of the entire study cohort were positive for spinal subdural haematoma. All the six “large” collections spread out from the lower spinal canal point (the sacral thecal cul-de-sac) to variable upper levels. Only two were seen to reach the cervical spine, along with one of the so-called “small collections” who was detected exclusively at this level. It followed that the higher majority of blood subdural collection involved the thoracolumbar portion of the spine rather than the cervical region.

**Table 1:** Spinal cord AHT pathological findings: evidence from neuroradiology (case series)

Study	Cases	Spinal cord level	Spinal cord injuries	Muscolo-skeletal spinal injuries	Imaging technique	Author’s statement
Rabbitt et al., 2020 [41]	47 AHT and 29 accidental head trauma (mean age and age interval are not given)	All levels	Spinal SDH was the only finding associated with a combination of RH (*p* = 0.001), non-contact head injury (*p* = 0.008) and AHT diagnosis (*p* < 0.05).	42% of AHT had ligament injury.	MRI	Spinal injury is seen in most AHT children and might be clinically and forensically valuable.
Jauregui et al., 2019 [42]	116 abused and 22,076 non abused (ma 2.1 and 13.9, respectively)	All levels	No increased risk of spinal cord injury in abused compared with non-abused (OR = 1.51).	Abused have increased risk of vertebral fractures at thoraco (OR = 2.97) and lumbar (OR = 1.67) level.	Not reported	Abused with spinal cord/vertebral injury are less likely to be admitted with cervical vertebra column fractures compared with non-abused children (OR = 0.51)
Henry et al., 2018 [43]	74 AHT and 14 accidental head trauma (<2 y)	Cervical	23% of AHT and 1.3% of accidental head trauma had spinal extra-axial haemorrhage.	9% of AHT and 6% of accidental head trauma had ligamentous injury.	CT MRI	AHT are at increased risk of cervical injuries.
Oh et al., 2017 [44]	91 abusive trauma (<9 y, ma 6 mo)	Cervical	2/91 had SDH, 4 had spinal cord injuries.	13/91 had ligamentous injuries, 22/91 had soft-tissue injuries.	MRI	In abused children, the rate of positive cervical MRI is up to 31%.
Baerg et al.,2017 [45]	53 AHT (<36 mo, ma 5 mo)	Cervical	1 case of cord injury with cord EDH and a case of an isolated cord EDH.	Ligamentous, vertebral artery shear injuries, atlantoccipital dissociation.	CT MRI	Small children with inflicted trauma had cervical spine injuries in around 15.1% of cases. Evaluation of them should include cervical spine imaging.
Jacob et al.,2016 [46]	89 AHT (<5 y, ma 9.1 mo)	Cervical	18% had SDH.	67% ligamentous 32% vertebral joint swelling.	MRI	The prevalence of cervical spine injuries in AHT children is high.
Kadom et al., 2014 [16]	38 AHT and 26 accidental head trauma and 10 undefined-head trauma (0.6–22.6 mo, ma 5.5 mo)	Cervical	2 children had spinal cord injuries.	27/74 had cervical soft-tissue injuries (data for single categories are not reported).	MRI	Spinal MRI does not discriminate AHT from accidental head trauma MRI can be helpful to distinguish traumatic from non-traumatic (but non-traumatic cases were not included in the study).
Choudhary et al., 2014 [47]	67 AHT and 46 accidental head trauma and 70 non-traumatic (all <48 months, ma 4 mo, 15 mo, 14 mo, respectively)	All levels	48% of AHT vs 2% of accidental head trauma had SDH (all in association with intracranial SDH). None of the non-traumatic had SDH.	78% of AHT vs 46% of accidental head trauma had ligamentous injuries. Nearly none (1%) of the abusive had MSSI	MRI	The statistically proven correlation between occipitocervical ligamentous injuries and intracranial findings (brain ischaemia) suggest upper occipitocervical spinal cord injuries leads to hypoxic-ischaemic encephalopathy.
Choudhary et al., 2012 [48]	67 AHT and 70 accidental head trauma (between 0 to 2 yo) who underwent CT/MRI of head and spinal cord	All levels	46% of AHT had SDH as compared with 1% of accidental head trauma. SDH finding is more frequent at thoracolumbar than cervical levels (63% vs 34%) (all in association with intracranial SDH).	Not mentioned.	CT MRI	Spinal SDH is statistically (*p* < 0.001) related to AHT.
Edelbauer et al., 2012 [49]	6 AHT and 12 non-traumatic (ma 3.3 and 2.5 mo, respectively)	All levels	Spinal SDH was seen in all AHT children from the cervical to the cauda equine.	No vertebral fractures.	US CT MRI RX	Spinal US should be part of the imaging examinations in case of suspected abuse.
Kemp et al.,2011 [35]	25 AHT with spinal injuries (1–48 mo)	All levels	Central cord injuries, compression, transection. Stroke, contusion, tethering.	10/12 of those with cervical lesions and 11/12 of those with thoracolumbar lesions had MSSI, the majority in association with spinal cord injuries.	RX CT MRI	Cervical spinal injuries are more frequent between younger infants (ma 5 mo). Thoracolumbar injuries are mainly seen in older children (13.5 mo). One of the cause of the delayed diagnosis are the unrecognized thoracolumbar injuries.
Koumellis et al., 2009 [50]	18 AHT (1–12 mo, ma 3 mo)	All levels	8/18 (44%) had spinal SDH (all had the same intensity of posterior fossa SDH and in 2 cases spinal collections were in continuity with intracranial collection).	2/18 spinal fractures on plan radiography.	CT MRI	There is a high incidence of unsuspected spinal SDH in AHT children. The location of it is commonly thoracolumbar rather than cervical.
Feldman et al., 1997 [40]	12 AHT (mean age and age interval are not given) 5/12 deceased (1.3–34.1 mo, ma 5.8 mo)	Cervical	MRI showed no cervical spinal cord injuries 4/5 had cervical spine bleeding at PM.	Not mentioned.	MRI	Routine cervical MRI is not convenient to identify cervical spinal cord injuries as well as to recognize abused babies.

*AHT* abusive head trauma, *CT* computed tomography, *yo* years old, *ma* mean age, *mo* months, *MRI* magnetic resonance, *MSSI* muscoloskeletal spinal injury, *PM* post mortem, *RH* retinal haemorrhages, *Rx radiography, SC* spinal cord, *SDH* subdural haematoma, *US* ultrasound scan

Tearing and laceration of blood vessels located in the spinal canal travelling along with the spinal nerves and ventral and dorsal nerve roots were supposed to be the primary source of spinal blood collection. Gruber et al. described a case of a 4-month-old boy brought to a trauma centre in respiratory arrest after being repeatedly shaken in 2008 [51]. After a T10-L1 subdural haematoma was seen on MRI, the source of the haemorrhage was intraoperatively identified within a lacerated radicular vein dorsal to the conus medullaris, which was coagulated and the bleeding stopped. The authors hypothesised that the connection between the thoracic and lumbar column is the pivot point in the shaking backward and forward body movements in the same manner of the cervical spine, and this can give a valuable explanation as to the thoracolumbar location of the haematoma.

Along with MRI and CT scan, ultrasound (US) examination was used by Edelbauer et al. (2012) to investigate the spinal cord in six AHT infants (mean age 3.3 ± 1.5 months) [49]. Spinal SDH was successfully seen, topographically extended from the cervical spine to the cauda equine as opposed to none of the 12 control.

In 2012, Choudhary et al. focused on the incidence of spinal subdural haemorrhage on imaging examination (MRI and CT) between 67 AHT babies, in comparison to 70 cases of accidental head trauma [48]. The cases were collected from an abusive head trauma registry, and no further information on the abuse assessment was given. In the AHT group spinal SDH accounted for about half of the cases (46%) as opposed to just above zero in those accidentally injured (1%). Cervical SDH was seen in 34% compared with subdural bleeding at thoracolumbar level in 63%. It was shown that abusive head trauma is statistically associated with subdural haematoma in spinal cord (*p* < 0.001) and is more frequently seen at the thoracolumbar region rather than the cervical level. Furthermore, Choudhary et al.’s work from 2014 confirmed the high proportion of SDH in 67 AHT cases (48%) as opposed to just 2% in the accidentally injured group in a comprehensive study of 46 babies (mean age 4 months) [47]. None of the 70 non-traumatic cases showed SDH.

Many studies focused on the MRI examination of the cervical level only. When Kadom et al. (2014) assessed the cervical MRI results from 38 AHT cases, only one had blood collection at subdural levels [16]. Similarly, Jacob et al. (2016) collected the cervical spinal cord MRI findings in 89 AHT infants (mean age 9.1 months) detecting an overall amount of SDH collections as low as 18% [46]. Finally, 53 AHT cases studied by Baerg et al. (2017) were all negative for spinal blood collection on MRI examination [45]. The study from Oh et al. (2017) analysed the overall results of imaging of the cervical spine in 503 abused children under the age of 9 years old. MRI was performed on 91 patients, and only two were positive for subdural blood collections [44]. In the study from Henry et al. (2018) on 74 AHT and 14 accidental injury head trauma children under the age of 2, who underwent cervical MRI or CT for causes other than motor vehicle crash, spinal extra-axial haemorrhage was detected in up to 23% in those with AHT as opposed to only 1.3% in those accidentally injured [43].

SDH in the spinal cord is commonly detected, when imaging investigation in AHT cases is performed on the whole length rather than part of the spinal cord. Agarwal et al. (2016) described a 6-month-old girl with intracranial bilateral SDH and retinal haemorrhages. MRI showed spinal haematoma extending from the thoracolumbar junction to the sacrum with a mass effect [52]. In 2019 Hong et al. reported a case of a 5-month-old boy suspect for abuse with bilateral intracranial SDH, and a subdural haematoma from T4 to L5 was seen at MRI examination [53]**.** As showed by Rabbitt et al.’s study from 2020 on 76 children who received spine MRI for identification of abuse, children with whole spine imaging were more likely to have spinal SDH (*p* = 0.03) compared with those with spinal cervical assessment only [41]. Unfortunately, 93% of abused babies were imaged at cervical and upper thoracic levels only. Although association between spine injury and abuse was not found, spinal subdural haemorrhage was the only finding associated with a combination of retinal haemorrhage (*p* = 0.01), non-contact head injury (*p* = 0.008) and a diagnosis of AHT (*p* < 0.05). Finally, when intracranial haemorrhage was analysed, it was shown to not be statistically associated with spinal SDH (*p* = 0.28).

#### Spinal ligamentous injuries

When spinal cord is studied through CT scan or MRI, one of the most recurrent features are changes in the soft-tissue apparatus, specifically in the ligamentous structure of the cervical column.

Ghatan et al. reported a case of a 24-day-old female victim of AHT [54]. MRI at cervical spinal cord showed ligamentous injury at occipitocervical junction, with atlantoaxial subluxation and narrowing of the spinal canal in 2002. Another case report from Bode et al. (2007) of a 8-month-old boy showed spinal ligamentous injuries at a lower level (disruption of the posterior ligament structure and cord contusion at T11–T12) [55]. In both the reports, it is not specified how the abuse was assessed. Kemp et al. (2011) published a comprehensive systematic review of 19 previous studies, and a total of 25 children (between 1 and 48 months of age) with the aim of identifying the clinical and radiological spinal cord features of abuse and all the children with a highly assured diagnosis of AHT who underwent spinal radiological examination (RMI, CT and RX) were included [35]. They found that the number with cervical lesions was as high as those with thoracolumbar lesions, accounting for 12 cases each. Both the cervical and the thoracolumbar injuries were mainly musculoskeletal, frequently in association with spinal cord involvement. In the cervical-lesion group 10 out of 12 had musculoskeletal injury, six of them with spinal cord compressions, transections, lacerations, stroke and parenchymal injury, while in the thoracolumbar-lesion group, the musculoskeletal injury accounted for 11/12, six of them with a spinal cord involvement (compression, contusion and tethering). Although the two groups had many aspects in common, those with lesions at the cervical level appeared younger as the majority of children were under 1 year of age as opposed to the thoracolumbar-injury group where the mean age was 13.5 months. In contrast with Feldman et al.’s statement that MRI should be performed only in the presence of spinal cord signs [40], the article from Kemp highlighted the mandatory role of MRI in order to prevent delayed recognition of spinal injuries.

When Choudhary et al.’s study from 2014 compared 67 AHT babies (mean age 4 months) to 46 accidental-injury and 70 additional cases who underwent MRI for causes other than trauma (mean age 15 and 14 months, respectively), spinal ligamentous injury appeared related to the abuse mechanism of trauma [47]. In those with AHT, ligamentous injuries accounted for 78% of the cases, compared with 46% in those accidental injuries and just over 0% in those with non-traumatic causes.

In 2014, Kadom et al. published a study on 74 children, 38 of them with abusive, 26 with accidental head trauma and 10 so-called “undefined-head trauma” who underwent brain and cervical MRI (mean age 5.5, 0.6 and 22.6 months, respectively). The AHT cohort was assessed through modified Duhaime criteria, an algorithm including injury type, history and associated findings used to classify each injury as inflicted or accidental [16, 56]. Overall, 27/74 had cervical soft-tissue injuries, but data on single categories were not given. The author stated the absence of a significant relationship between cervical spinal injuries and abusive head trauma and therefore suggests that MRI lacks the ability to discriminate between accidental and abusive head traumas. However, the precise rate of AHT and accidentally injured children suffering from cervical injury was not given. In more recent years, Jacob (2016) published a retrospective review on 89 AHT children under the age of 5 years (mean age 9.1 months) to identify the features of cervical spine on MRI [46]. Cervical spine injury was reported to be as high as 69%, mainly based on ligamentous alterations (67%) and vertebral joint swelling. Furthermore, Baerg et al. (2017) analysed MRI data from cervical spinal cord of 53 AHT children under the age of 36 months (mean age 5 months) [45]. The percentage with cervical spine injury was reported to be 8/56 while ligamentous injuries were seen in 2/8 (25%). Finally, when Oh et al. (2017) studied the results form 91 abused patients (under the age of 9 years old) with cervical MRI, 13 (14%) were positive for ligamentous injuries [44], according to Henry et al.’s study from 2018, where ligamentous injuries were up to 9% in AHT and 6% in those accidentally injured when cervical spinal cord is imaged by MRI or CT [43].

In conclusion, the incidence of spinal ligamentous injuries in AHT varies from 9 to 78%. As opposed to spinal subdural blood collections (mainly seen at thoracolumbar level), cervical level seems to be the ideal topographic location for detecting ligamentous injuries due to abuse trauma. However, the correlation between abuse and changes in ligamentous structures appeared to be not statistically proven, and even if soft-tissue lesions can strengthen the suspect of abuse, the finding alone is not sufficient to lead the diagnosis of abusive head trauma.

#### Additional radiological findings

According to the different modalities, abuse can happen as it is easy to suppose spinal structure is involved in many ways showing a wide range of additional features. Here below are reported those available from the current scientific knowledge i.e. cord parenchyma and spinal bone structure injuries.

Vertebral fractures were found in 2/18 cases in Koumellis et al.’s study from 2009 at the level of the thoracic spine imaged by plan radiography [50]. In Kemp et al.’s study (2011), those with cervical lesions (8/12) had spinal cord involvement (central cord injury, spinal cord compression and transection) [35], and just one case had vertebral arterial obstruction and stroke. In the group of musculoskeletal lesions (10/12), skeletal injuries varied between Hangman’s fracture at C2/C3, anterolisthesis, compression fracture of vertebral body and bilateral pedicle fractures. Between those with lesions at thoracolumbar level, 6/12 had spinal cord involvement with compression, contusion and tethering, and nine out of 12 had fracture dislocations, and three had compression of the vertebral body.

Joint swelling in 32% of AHT cases was reported by Jacob et al. (2016) [46]. They also highlighted that bone marrow oedema is usually seen in older children (mean age 14.9 months, *p* = 0.028) while capsular injury is commonly seen in younger children (mean age 5.5 months, *p* = 0.006).

A spinal cord transection was detected by CT at T4 level in association with a distraction fracture of the spine on MRI in Brink et al.’s case (2017) of a 5-week-old boy [57]; the mother confessed to have grabbed him from the ankles and hit his back against a solid surface.

Probably the most comprehensive study was the one from Jauregui et al. (2019) who retrospectively reviewed 22,192 children with spinal column fractures or spinal cord injuries [42]. Patients were identified from Kids’ Inpatients Database (KID) using ICD-9-CM diagnosis (cervical, thoracic, lumbar vertebral fracture and spinal cord injury). One hundred and sixteen cases had a documented diagnosis of abuse and were shown to be at higher risk of thoracic (OR = 2.57) and lumbar (OR = 1.67) vertebral fractures as compared with non-abused patients. Additionally, abused patients were significantly less likely to be admitted with cervical column fractures than non-abused patients (OR = 0.51). Overall, no increased risk of spinal cord injury in abused compared with non-abused cases was seen. In conclusion, although the findings are commonly seen in abused babies, they appeared to be highly specific for the mechanism of trauma and so not indicative for abuse or accidental mechanism of trauma**.**

### Spinal cord AHT: evidence from neuropathology

#### Spinal blood collections

The first recorded study examining the neuropathology of spinal cord injury related to AHT was carried out in 1989 by Hadley et al. when they studied 13 infants (mean age 3 months) who died of confessed shaking without evidence of head impact trauma [28] (Table 2). Six of them underwent post mortem examination, and spinal cords were examined. All except one of the autopsied children showed injuries in the spinal cord, five had epidural haematoma and four had subdural haematoma at the cervicomedullary junction along with contusions of the ventral high cervical levels. All the autopsied children who had SDH presented with cerebral contusions, swelling and herniations. Hadley concluded that spinal injury at the high cervical cord level can contribute to the dramatic outcome of shaking without direct cranial impact. He also underlined the very young age of the studied babies, suggesting infants are more susceptible to injury from shaking.

**Table 2:** Spinal cord AHT pathological findings: evidence from neuropathology (case series)

Study	Cases	Spinal cord level	Spinal cord injuries	Muscolo-skeletal spinal injuries	Author’s statement
Serinelli et al., 2017 [58]	51 homicide victims (42 AHT) (<3 yo)	All levels	Spinal cord injuries at toracholumbar (33%) > lumbosacral 27.5 > cervical 15.5%.	Not mentioned.	When considering the distribution of SC injuries (EDH, SDH, SAH), the thoracic SC was the most frequently involved area of the SC.
Brennan et al., 2009 [36]	41 AHT and 11 accidental head trauma (<2 yo)	Cervical	71% AHT had primary cervical spinal cord injuries: 72% parenchymal, 83% meningeal haemorrhages, 55% nerve roots (avulsion, haemorrhages).	21% among those AHT with spinal cord injuries had soft-tissue injuries.	Cervical spinal cord injury is a frequent but not universal finding in AHT. Parenchymal/dorsal nerve roots injuries can occur without ligamentous cervical injuries.
Geddes et al., 2001 (II) [59]	37 AHT (<9 mo, ma 2.4 mo) 14 non-traumatic (<11 mo, ma 3 mo)	Cervical	3/28 AHT showed βAPP positivity in cervical cord and/or dorsal nerve roots 8/28 AHT showed βAPP positivity in the lower pons and medulla 0/14 non-traumatic had βAPP positivity.	Not mentioned.	The predominant histological abnormality in AHT is diffuse hypoxic brain damage not axonal injury.
Geddes et al.,2001 (I) [32]	53 AHT (0.5–97 mo, ma 4 mo)	Cervical	3/53 had EDH 3/53 AHT showed βAPP positivity in cervical cord 8 AHT showed βAPP positivity in the lower pons and medulla.	Not mentioned.	AHT damage is age-related: infants (ma 2–3 mo) had thin bilateral intracranial SDH and higher incidence of skull fractures; AI is seen in craniocervical junction. Children >1 yo had larger intracranial SDH collection and higher incidence of extracranial damage: AI is seen in hemispheric with matter.
Saternus et al., 2000 [31]	4 AHT (shaking only) (3 autopsied and 1 survived) (4–30 mo, ma 13.7 mo)	Cervical	1/3 had cervical EDH (dorsal, C2/C3–C5/T2). Survived children had blood-stayed CSF.	No skeletal fractures were found at skeletal survey in 4/4 spine radiographs 2/3 had cervical soft-tissue injuries.	In every case of child autopsy, it is mandatory to look at cervical spinal cord.
Shannon et al., 1998 [60]	13 AHT (shaken only) (<2 yo, ma 5 mo) 7 hypoxia, 6 sudden asphyxia children	Cervical	7/11 AHT showed βAPP positivity in cervical cord and in spinal nerve roots, as opposed to none in the control groups.	Not mentioned.	Cerebral axonal injury is common in shaken babies and may be due in part by hypoxic/ischaemic mechanism. Cervical cord inj. is also common and cannot be attributed to HIE, so a traumatic mechanism may play a crucial role.
Feldman et al., 1997 [40]	5 AHT (1.3–34.1 mo, ma 5.8 mo)	Cervical	1/5 had SDH at the upper cervical cord, in association with cranial SDH 3/5 had SAH at the cervical cord level, in association with similar intracranial findings.	No skeletal fractures were found at skeletal survey in 12/12 spine radiographs.	Routine cervical MRI is not convenient to identify cervical spinal cord injuries as well as to recognize abused babies.
Hadley et al., 1989 [28]	13 AHT (shaken only) (ma 3 mo) (6 autopsied)	Cervical	5/6 had EDH at cervicomedullary junction 4/6 had SDH at cervicomedullary junction 4/6 had ventral spinal contusions at high cervical levels.	Not mentioned.	Haemorrhages and contusions of the high cervical cord may contribute to morbidity and mortality in shaken baby syndrome.

*AHT* abusive head trauma, *βAPP* β amyloid precursor protein, *EDH* epidural haematoma, *yo* years old, *ma* mean age, *mo* months old, *MRI* magnetic resonance, *SAH* Subarachnoid haematoma, *SDH* subdural haematoma

Eight years later, Feldman et al. (1997) focused on spinal cord injuries in five AHT autopsied children (mean age 5.8 months) enrolled through the child protection team [40]. The diagnosis of inflicted head injury was then corroborated by the infant’s attending physician. In only one case, subdural blood collection was seen in the cervical spinal cord, while 3/5 showed subarachnoid bleeding. Both the subdural and the subarachnoid haemorrhages were seen in association with similar intracranial findings, and subdural haematoma was in clear continuity with the spinal one.

In the three autopsied children reported by Saternus et al. (2000) (mean age 16 months), the AHT diagnoses were assumed from the history taken in the police notes in association with the intracranial subdural haematoma and the absence of head impact signs [31]. One had epidural haemorrhage at the cervical level, and none of the cases had subdural blood collections in the spinal cord. The cervical spine showed intervertebral disc rupture and blood collections in the soft tissue in 2/3 patients. Similarly, when Geddes et al. (2001) performed a comprehensive retrospective study in order to identify the neuropathological changes in AHT children, the only spinal blood collection was the epidural haematoma seen in three cases [32]. In an attempt to determine specifically the neuropathological findings in the cervical spinal cord of AHT, Brennan et al. (2009) reported the outcomes from 41 children who underwent cervical examination at post mortem and assessed to have died from AHT by the chief medical examiner [36]. A very high proportion of them showed meningeal haemorrhages (83%) (epidural, intradural, subdural and/or subarachnoid, in an unspecified proportion) and parenchymal lesions such as contusions, lacerations and transections (72%). Additionally, nerve root avulsions and dorsal root ganglion were seen in slightly more than a half (55%). None of the children had vertebral fractures, and only one fifth (21%) had soft-tissue injuries in the neck.

All the above-mentioned studies focused on cervical level spinal cord. Following the evidence from neuroradiological investigation of spinal cord, showing thoracolumbar level is the common location for spinal blood collection, is it possible that spinal cord injuries were overlooked and that the real incidence of them from neuropathology investigation is underestimated? For instance when homicide victims from physical abuse under the age of 3 years old were studied by Serenelli et al. in 2017, the majority of spinal cord lesions were at spinal level lower than cervical [58]. In this cohort of 51 children (42 AHT), the most common finding was SDH across the spinal cord. Spinal cord injuries at a thoracolumbar location accounted for the majority of the cases (33.3%) as compared with the lumbosacral area (27.5%) and the cervical level (15.5%). Thoracic location appeared more frequent in infants, and the correlation was statistically proven (*p* = 0.048).

### What is the source of spinal SDH?

**Fig. 1:**
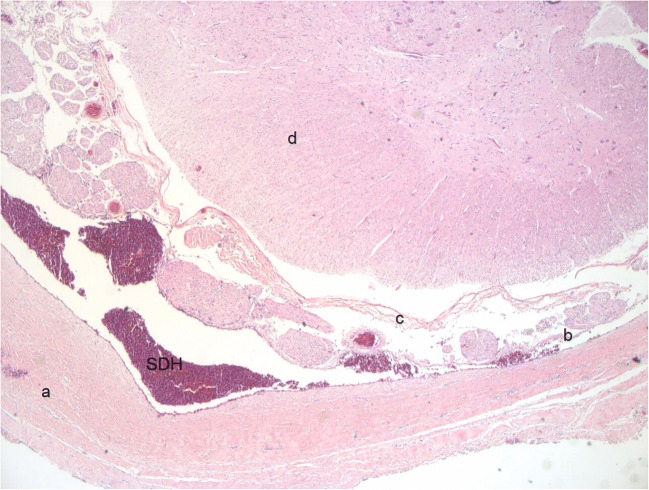
Hematoxylin-eosin-stained histological sections of thoracic spinal cord with recent SDH in a 5-month-old female victim of abusive head injury (original magnification ×25). a: dura mater, b: arachnoid, c: pia mater, d: spinal cord, SDH subdural haematoma

**Fig. 2:**
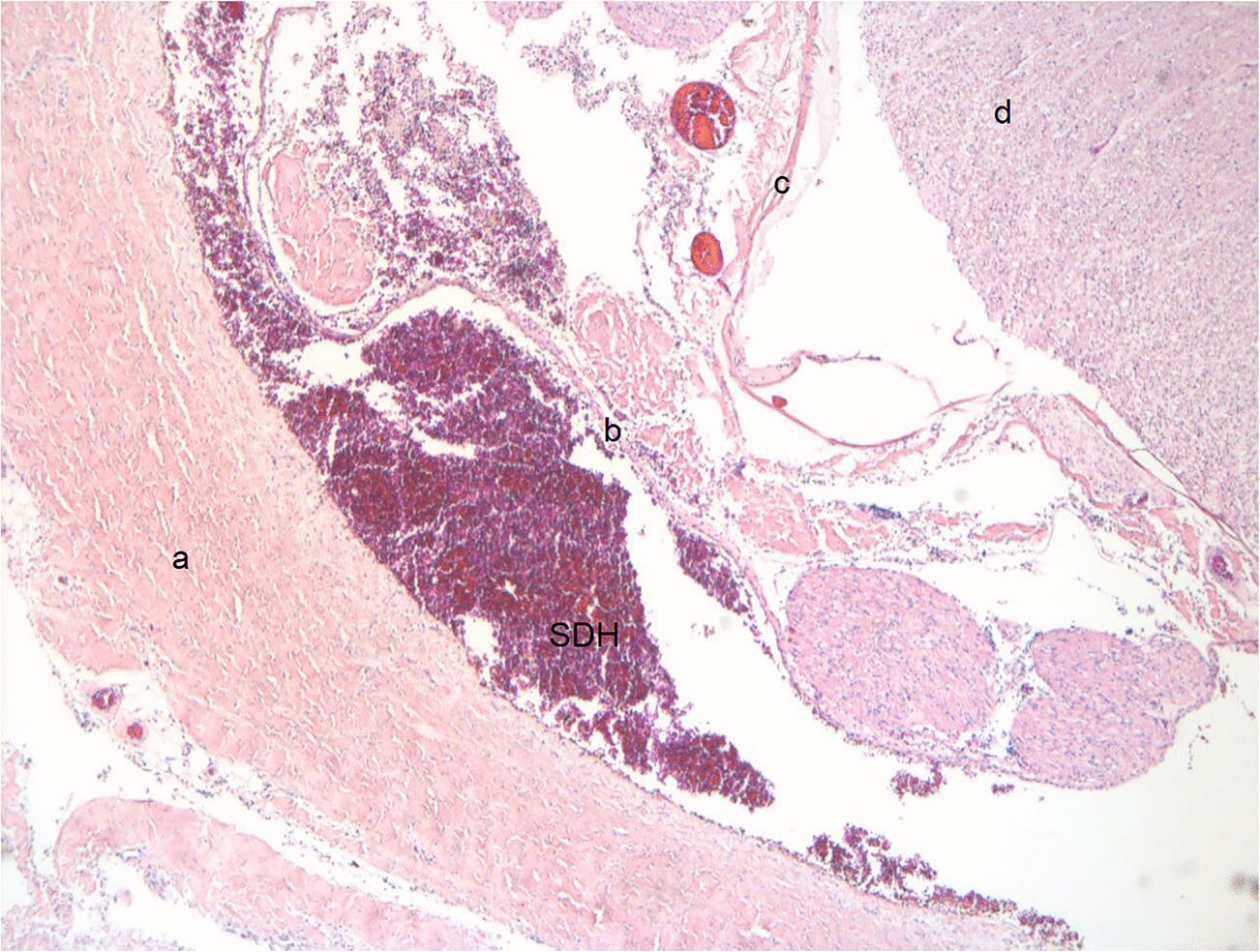
Hematoxylin-eosin-stained histological sections of spinal cord thoracic segment in a 5-month-old female victim of abusive head injury (original magnification ×50) a: dura mater, b: arachnoid, c: pia mater, d: spinal cord, SDH subdural haematoma

**Fig. 3:**
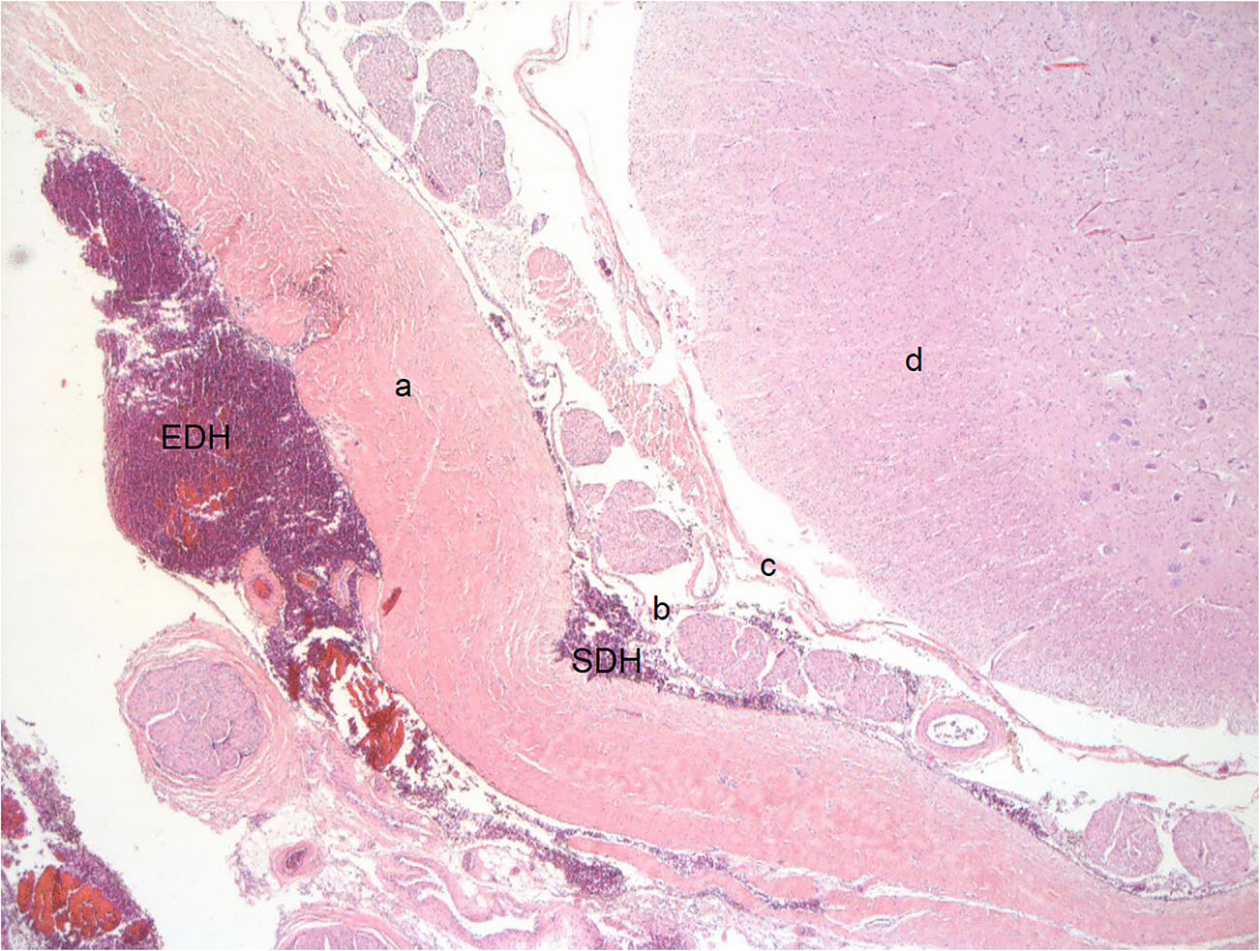
Hematoxylin-eosin-stained histological sections of cervical spinal cord with extradural and subdural haematoma in a 5-month-old female victim of abusive head injury (original magnification ×25) a: dura mater, b: arachnoid, c: pia mater, d: spinal cord, SN spinal nerves, EDH extradural haematoma, SDH subdural haematoma

The anatomical structure of the spinal cord was proposed as the reason for the thoracolumbar distribution of subdural collections [50]. The blood would flow from the posterior fossa into the spinal canal, collecting at the thoracolumbar region, where “a natural convexity in the supine position is present”. In our review, all the cases in the radiological and pathological investigations showed spinal SDH in association with intracranial SDH which was an inclusion criteria in only 5/9 and 1/9 articles (Tables 3 and 4) (Figs. 1, 2 and 3).

**Table 3:** Spinal cord AHT pathological findings: evidence from neuroradiology (case series and case reports). Is intracranial SDH associated?

Study	Cases	Spinal cord injuries	Intracranial SDH in AHT cases	Is intracranial SDH inclusion criteria?
Rabbitt et al., 2020 [41]	47 AHT and 29 accidental head trauma (mean age and age interval are not given)	Spinal SDH was the only finding associated with a combination of RH (*p* = 0.001), non-contact head injury (*p* = 0.008) and AHT diagnosis (*p* < 0.05).	Yes (fifty-nine children (78%) who received an MRI spine had an intracranial haemorrhage on MRI brain. Of these, 11 (19%) had co-occurring spinal SDH).	No
Hong et al., 2019 [53]	Case report of a 5-month-old boy	Spinal SDH from T4 to L5	Yes	Yes
Henry et al., 2018 [43]	74 AHT and 14 accidental head trauma (<2 y)	23% of AHT and 1.3% of accidental head trauma had extra-axial haemorrhage	Yes (87%)	No
Oh et al., 2017 [44]	91 abuse trauma (<9 y, ma 6 mo)	2/91 had SDH, 4 had spinal cord injuries	Not reported	Yes, but not exclusively
Agarwal et al., 2016 [52]	Case report of a 6-month-old girl	Spinal SDH from thoracolumbar junction to the sacrum with mild mass effect	Yes	Yes
Jacob et al., 2016 [46]	89 AHT (<5 y, ma 9.1 mo)	Overall spinal cord injuries: 69% (67% ligamentous, 18% SDH)	Yes	No
Kandom et al., 2014 [16]	38 AHT and 26 accidental head trauma and 10 undefined-head trauma (0.6 mo–22.6 mo, ma 5.5 mo)	1 child had intrathecal haemorrhage 2 children had spinal cord injuries	Yes	Yes
Choundhary et al., 2014 [47]	67 AHT and 46 accidental head trauma and 70 non-traumatic (all <48 months, ma 4 mo, 15 mo, 14 mo, respectively)	48% of AHT vs 2% of accidental head trauma had SDH (all in association with intracranial SDH). None of the non-traumatic had SDH.	Yes	No
Choundhary et al., 2012 [48]	67 AHT and 70 accidental head trauma (between 0 and 2 y) who underwent CT/MRI of head and spinal cord	46% of AHT had SDH as compared with 1% of accidental head trauma. SDH finding is more frequent at thoracolumbar than cervical levels (63% vs 24%) (all in association with intracranial SDH). Accidental head trauma	Yes	No
Edelbauer et al., 2012 [49]	6 AHT and 12 non-traumatic (ma 3.3 and 2.5 months, respectively)	Spinal SDH was seen in all AHT children from the cervical to the cauda equina	Yes	No
Gruber et al., 2008 [51]	Case report of a 4-month-old boy	T10-L1 subdural haematoma	Yes	No
Koumellis et al., 2009 [50]	18 AHT (ma 3 mo, 1–12 mo)	8/18 (44%) had spinal SDH (all had the same intensity of posterior fossa SDH, and in 2 cases spinal collections were in continuity with intracranial collection)	Yes	No
Ghatan et al., 2002 [54]	24-day-old girl	MRI showed ligamentous injury at occipitocervical junction, with atlantoaxial subluxation and narrowing of the spinal canal	Yes	No
Feldman et al., 1997 [40]	12 AHT (mean age and age interval are not given) 5/12 deceased (1.3–34.1 mo, ma 5.8 mo)	MRI showed no cervical spinal cord injuries (0/12) 4/5 had cervical spine bleeding at PM	Yes	Yes
Diamond et al., 1994 [39]	12-month-old girl	The MRI scan showed a T12-L3 pre-spinal mass possibly of haemorrhagic nature and tethered cord.	No	No

*AHT* abusive head trauma, *yo* years old, *ma* mean age, *mo* months, *MRI* magnetic resonance, *PM* post mortem, *SDH* subdural haematoma

**Table 4:** Spinal cord AHT pathological findings: evidence from neuropathology (case series and case reports). Is intracranial SDH associated?

Study	Cases	Spinal cord injuries	Intracranial SDH in AHT cases	Is intracranial SDH inclusion criteria?
Serinelli et al., 2017 [58]	51 homicide victims (42 AHT) (<3 yo)	Spinal cord injuries at toracholumbar (33%) > lumbosacral 27.5 > cervical 15.5%	Yes (82.4%)	No
Brennan et al., 2009 [36]	41 AHT and 11 accidental head trauma (<2 yo)	71% AHT had primary cervical spinal cord injuries: 72% parenchymal, 83% meningeal haemorrhages, 55% nerve roots (avulsion, haemorrhages).	Yes (92%)	No
Geddes et al., 2001 (II) [59]	37 AHT (<9 mo, ma 2.4 mo) 14 non-traumatic (<11 mo, ma 3 mo)	3/28 AHT showed βAPP positivity in cervical cord and/or dorsal nerve roots 8/28 AHT showed βAPP positivity in the lower pons and medulla 0/14 non-traumatic had βAPP positivity.	Yes (81%)	No
Geddes et al., 2001 (I) [32]	53 AHT (0.5–97 mo, ma 4 mo)	3/53 had EDH 3/53 AHT showed βAPP positivity in cervical cord 8 AHT showed βAPP positivity in the lower pons and medulla.	Yes (81%)	No
Saternus et al., 2000 [31]	4 AHT (shaking only) (3 autopsied and 1 survived) (4–30 mo, ma 13.7 mo)	1/3 had cervical EDH (dorsal, C2/C3–C5/T2) Survived children had blood-stained CSF.	Yes (50%)	No
Shannon et al., 1998 [60]	13 AHT (shaken only) (<2 yo, ma 5 mo) 7 hypoxia, 6 sudden asphyxia- children.	7/11 AHT showed βAPP positivity in cervical cord and in spinal nerve roots, as opposed to none of the control groups.	Yes	No
Feldman et al., 1997 [40]	5 AHT (1.3–34.1 mo, ma 5.8 mo)	1/5 had SDH at the upper cervical cord, in continuity with cranial SDH 3/5 had SAH at the cervical cord level, in association with similar intracranial findings	Yes	Yes
Hadley et al., 1989 [28]	13 AHT (shaken only) (ma 3 mo) (6 autopsied)	5/6 had EDH at cervicomedullary junction 4/6 had SDH at cervicomedullary junction 4/6 had ventral spinal contusions at high cervical levels	Yes	No

*AHT* abusive head trauma, *βAPP* β amyloid precursor protein, *EDH* epidural haematoma, *yo* years old, *ma* mean age, *mo* months old, *MRI* magnetic resonance, *SAH* subarachnoid haematoma, *SDH* subdural haematoma

On the other hand, as recently shown by Rabbitt et al. (2019), spinal SDH in children evaluated for abusive head trauma is not commonly associated with intracranial haemorrhage (*p* = 0.28) [41]. The small size of the intracranial bleeding and the lack of SDH in the posterior fossa compartment are not consistent with the intracranial source of spinal blood subdual collections. Given the close contact of the subdural and subarachnoid sheets is easy to suppose that a small quantity of blood leaking from upper level is not enough to separate the two sheets and to collect downwards at the thoracolumbar level. In his case report, Gruber et al. (2008) described the origin of bleeding as primary in the spinal cord, intraoperatively seen as a lacerated radicular vein [51]. According to the finding, the author suggested the blood vessels travelling along with the spinal nerve roots are the source of the spinal subdural haemorrhages.

Anatomically, the spinal cord is surrounded by the meninges, in the same way as the brain but with some differences. The dura mater is composed of only one sheet which is the direct continuation of the inner meningeal layer of the cranial dura when the outer layer of intracranial dura mater ceases at the foramen magnum. The spinal dura mater is firmly attached to the circumference of the foramen magnum, to the second and third cervical vertebrae and with the posterior longitudinal ligaments as well. The sheath of dura mater is much larger than is necessary for the accommodation of its contents, and its size is greater in the cervical and lumbar regions than in the thoracic. The epidural space contains a plexus of veins, while the subdural cavity is not actual, but it is a virtual space as the dura is in close contact with the arachnoid. Therefore, it is possible that a large intracranial subdural haematoma may have enough volume and weight to force itself through the virtual subdural space and present as a spinal cord subdural haematoma. But it is less likely that a small mainly intracranial SDH (commonly seen in AHT) can travel through different compartments to reach the spinal cord. Both the dura and the arachnoid surround the spinal nerves at the level of their entrance in the spinal cord. The pia and arachnoid membranes continue along with the spinal nerve roots as they leave the spinal cord and exit through the intervertebral foramina, where they blend with the perineurium of the spinal nerves [61, 62]. Arterial and venous vessels run along the surface of the spinal cord, between the arachnoid and pia mater. The latter is composed of collagen and reticular fibres which wraps the surface of spinal cord, while collagen fibres are external and form bundles with the above arachnoid; it is in this virtual space where vessels are found [63].

One possible source of the spinal cord SDH is the radicular veins which run along nerve roots, and then the spinal cord surface needs to penetrate the arachnoid membrane in order to reach the subarachnoid location, resulting in an area of weakness when exposed to a high energy trauma such as that in shaking. The laceration of radicular veins at the point of passage from subdural to subarachnoid space could explain the blood collection in the spinal subdural space.

The hypothesis of primary damage in the spinal nerve roots is supported by further evidence, and damage in the nerve roots especially the dorsal nerve roots has been detected [51]. Brennan et al. reported the frequency of nerve roots avulsion and dorsal root ganglion haemorrhages as up to 55% between AHT children [36]. Likewise, the βAPP immunohistochemistry examinations were positive in the nerve roots of abused babies as compared with the control group where no expression was detectable [59, 60]. It is well known that spinal nerve roots are the site of CSF absorption, and so they are surrounded by a high density vein vessel mesh and therefore are prone to bleeding [1]. An association of high intracranial pressure and vessel damage due to hypoxic endothelial damage has been suggested as the cause of SDH. It is possible that the same mechanisms play a role in spinal cord bleeding [4]. The primary spinal source of blood collection is also supported by the increasing evidence that the thoracolumbar level is frequently involved in cases of spinal trauma due to abuse, such as Jauregui et al.’s observation of increased risk of thoracic (OR = 2.75) and lumbar (OR = 1.67) vertebral fractures in his cohort of 116 abused children [42]. In a recent study on 51 homicide victims, the frequency of thoracic and lumbar spinal injuries was reported around 30% as compared with just 15.5% at cervical level [58]. Consequently, the side of maximum forces could be thoracolumbar rather than the cervical as the thoracic spine with the ribcage provides another valuable pivot point at the level of its articulation with the lumbar spine. Detecting the primary source of blood collection is of great interest to clarify where the trauma forces acted and therefore to better understand the trauma mechanism. Further neuropathological studies looking at the whole spinal cord and spinal nerve roots are needed to solve the issue.

#### Spinal cord (parenchymal) injuries

**Fig. 4:**
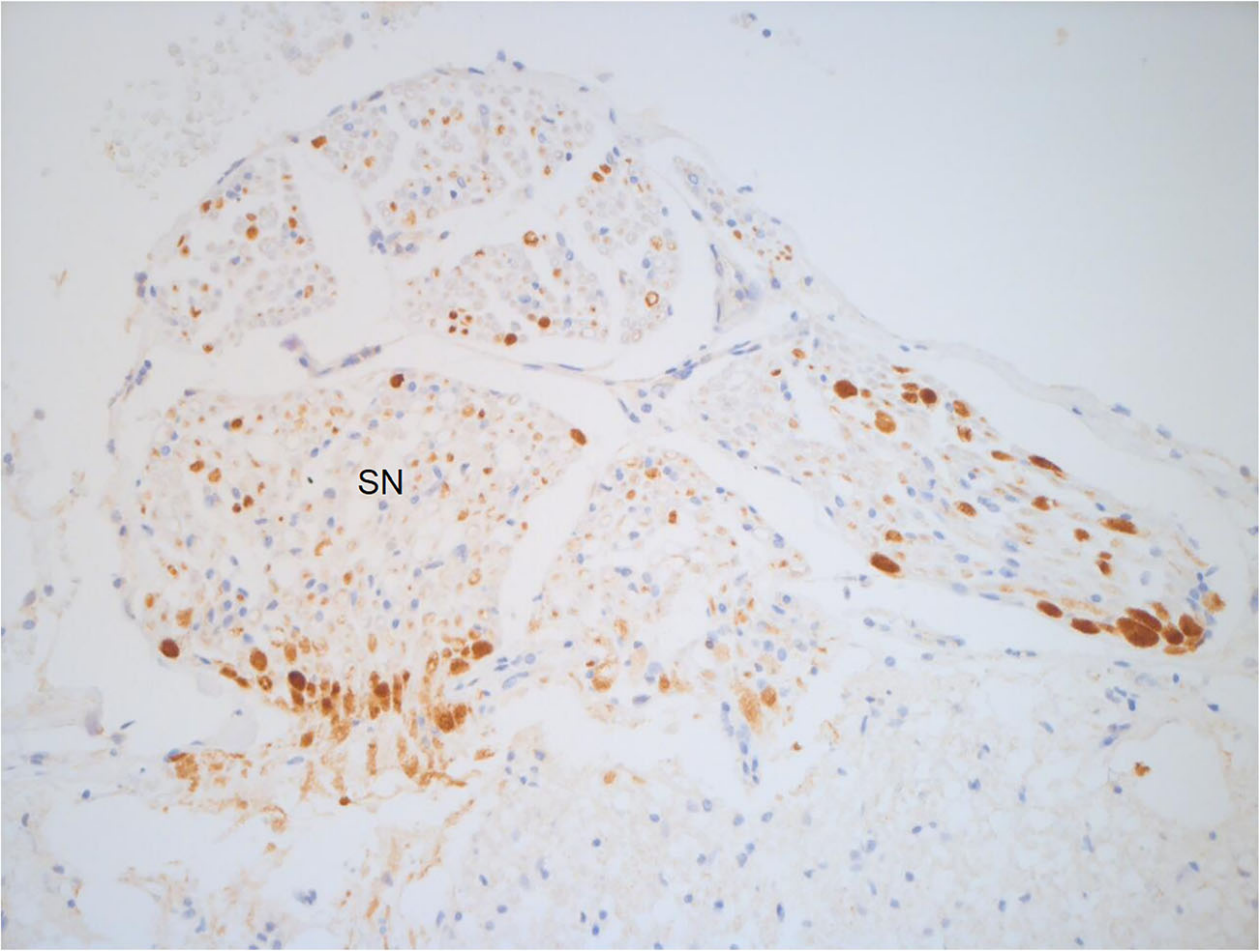
βAPP showing axonal injury in the posterior nerve roots in the cervical segment in a 4-week-old female victim of abusive head injury (original magnification ×25). SN spinal nerves

**Fig. 5:**
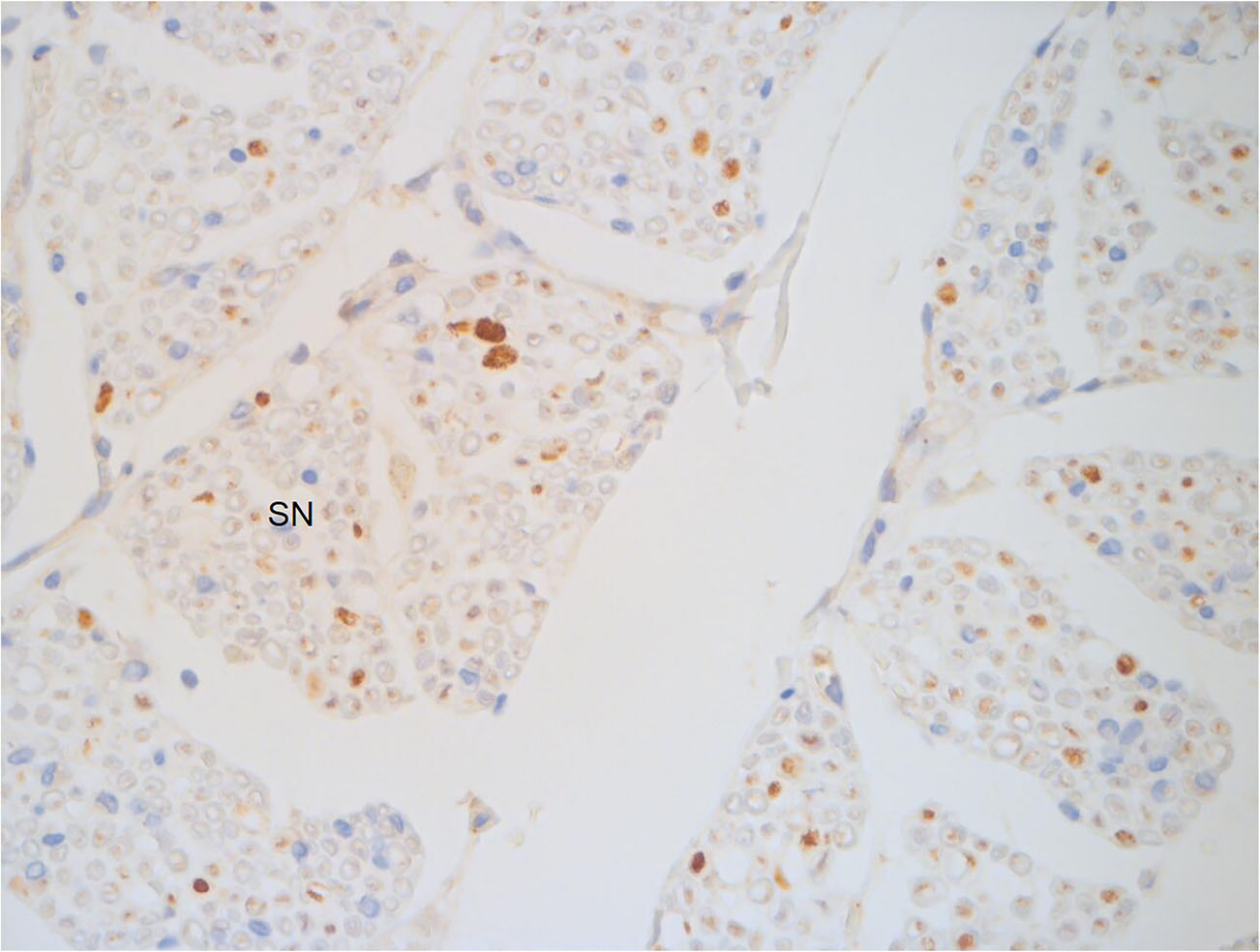
βAPP in the posterior nerve roots in cervical segment in a 4-week-old female victim of abusive head injury (original magnification ×50). SN spinal nerves

When Hadley et al. (1989) studied six AHT cases at post mortem, four had spinal contusions [28]. Twenty years later, Brennan et al. (2009) confirmed the recurrent involvement of spinal cord injury in abuse following the observation of parenchymal lesions in up to 72% of his cohort of 41 abused children [36]. Parenchymal lesions have been studied mainly through histology (Figs. 4 and 5). The first histological documentation of spinal injury in shaken babies was from Shannon et al. (1998) when fourteen cases (mean age 5 months) of witnessed, confessed or corroborated shaking without skull fractures underwent CD68 and βAPP immunohistochemistry [60]. Cervical spinal cord showed βAPP-positive axons in 7 out of 11 cases, along with the spinal nerve roots (especially in the glial head). On the contrary, none of the control group cases (death from hypoxic-ischaemic encephalopathy and asphyxia) showed positivity for βAPP staining in the same structure, and the author hypothesised that βAPP positivity in the white matter tracts of cervical spinal cord may be associated with shaking. Shannon also studied the medulla, midbrain and cerebral white matters, where no differences were seen between the two groups.

When Geddes et al. (2001) performed a comprehensive retrospective study in order to identify the neuropathological changes in AHT children, the findings were remarkable [32]. The study cohort was comprised of 53 AHT children (37 infants and 16 toddlers) retrospectively collected and corroborated according to diagnostic criteria proposed by the author based on perpetrator’s confession/conviction and extra-cranial injuries. On histology, 8 out of the 53 showed localized βAPP-axonal positivity in the corticospinal tracts of the lower brainstem (lower pons and medulla) as well as in cervical cord roots in three additional cases. The findings were explained by the author as the result of stretching forces acting on the corticospinal tract in the lower brainstem which may lead to apnoea and hypoxic-ischaemic damage. In the same year, Geddes et al. published another article where the same results from the 37 infants were compared with a control group of 14 infants who died from causes other than traumatic [59]. Three out of the 28 AHT cases immunologically stained were positive for βAPP in cervical cord and/or dorsal nerve roots, while eight showed βAPP positivity in the lower pons and medulla. None of the controls was positive for βAPP in the brain stem and spinal cord. Although the current knowledge on spinal cord changes in association to abuse need to be better investigated, is it possible for the time being to suppose that parenchymal injuries are not specific for the mechanism of trauma but possibly useful to understand the pathological mechanisms following abusive traumas?

### Effect of age on spinal cord injuries in AHT

It is well known that a disproportionately larger head in children is supported by weaker and more lax cervical muscle and ligaments than in adults [64]. What appears peculiar is the topical distribution of spinal injuries, which seems to be age-related. Cervical spinal injuries were seen more frequently in younger infants (age range 1–48 months, median age 5 months), while thoracolumbar was more frequent in older infants (age range 6–16 months, median age 13.5 months) [35]. According to the current literature, young children tend to injure the upper cervical spine at the craniocervical junction to the C3 spinal level [64]. As previously reported, infants have larger heads in proportion to their body and a more horizontal vertebral facet, allowing a higher degree of freedom in motion. When children grow, injuries are seen mainly in the lower level reaching the adult proportion at about 8–10 years. It has been proven that upper cervical spine injury (C1–C4), cervical fracture and spinal cord injury, spinal cord injury without radiographic abnormality (SCIWORA) and dislocation show a downward trend with increasing age [65]. As such, the pathophysiology of trauma could also be different. If younger children have craniocervical junction injury, which leads to hypoxic-ischaemic brain injury, older babies can a have major incidence of extracranial injury points such as at thoracolumbar spinal cord levels. It follows that radiological examination of the spinal cord should be routinely performed at all levels, particularly when the suspected abuse involves children older than 1 year of age.

Intracranial subdual haematoma is a common finding in neonates. When 101 term neonates were imaged by MRI within the first 72 h of life, the incidence of SDH was as high as 46%, the topical distribution involving both supra and infra tentorial compartments [66]. It has been suggested that SDH in a child over the age of 1 month should not be attributed completely to birth trauma [67]. Spinal cord haemorrhage in newborns was studied by Vlasyuk et al. in 2013 when he analysed 14 premature still-born babies with intraventricular haemorrhages. All cases with grade III were accompanied by drops of fluid in the subarachnoid space of the cervical dorsal and lumbar parts of the spinal cord [68]. No other cases of spinal blood in newborns are reported in the literature. Similarly, intracranial haemorrhage can be seen in newborns lacking vitamin K in association with cerebral oedema and can mimic abuse. Brain infection and spontaneous intracranial haemorrhages linked with diastasis may also mimic AHT as oedema, and SDH can be present on neuroimaging in cases of encephalopathy.

The clinical manifestation is also age-related. Children under 1 year of age present with impaired consciousness and respiratory distress while older patients have spinal deformity and focal neurological signs [35].

Infants have thin bilateral SDH and higher incidence of skull fracture, while children older than 1 year of age had larger intracranial SDH collections and a higher incidence of extracranial damage, suggesting that the head and craniocervical junction are the main injury points in infants, but not in older children [32]. It could be supposed that shaking acts on different levels depending on the age of the victim.

## Summary and conclusion

Forensic pathologist is usually asked to give a reason for the cause and mechanism of injuries detected through a wide-ranging examination. Then, abusive head trauma has been a source of interest for many years, and much effort has been spent in order to identify the characteristic features associated with it. Being a medical diagnosis AHT needs to be integrated with non-medical evidence to answer legal questions of actus reus (guilty act) or mens rea (guilty mind), and scientific evidence has the main role in providing an answer to the medical question of “whether an infant’s injuries were most likely caused by abuse or could they be plausibly explained by a hypothetical alternative” [69].

Spinal cord injuries could play a role in supporting the challenging AHT diagnosis when investigated both radiologically at time of admission and neuropathologically at post mortem examination. According to a recently published consensus statement supported by multiple societies, spinal cord MRI assessment of all spinal levels is now recommended in cases of suspected child abuse [69].

The vast majority of spinal cord assessment in cases of abuse has been obtained by MRI in cases of AHT variable incidence from 4.3 to 84.3% according to the different institutions [43, 69]. MRI has been shown to be an effective method of diagnosis in the paediatric population, because of the anatomical conformation of the spinal cord (made of cartilage in many vertebral components) and preferable to CT because of its high sensitivity for ligament injury [70].

One of the common features associated to abusive head trauma is subdural haemorrhage in the spinal cord which is poorly investigated compared with widely studied intracranial subdural haemorrhage. This could be due to limited literature on the incidence of spinal findings in AHT cases, non-specificity of clinical signs predicting the risk of spinal peculiar injury, logistical issues, high cost of imaging the entire spine, limited spinal stability and limited understanding of forensic markers [69].

Peculiarly, our results showed the more frequent location is thoracolumbar level rather than cervical. This data has major implication for the forensic pathologist as the suspect of abuse could not be completely ruled out without an investigation on the whole length of the spinal cord.

The origin of spinal blood collections is most likely the traumatic damage to the radicular veins which run close to the spinal nerve roots; both of which are probably damaged in movement of spinal cord within the spinal column following excessive forward and backward movements of the head and neck commonly encountered in AHT.

Another important feature associated to abuse is spinal ligamentous injury. Although it has been reported an incidence up to 78% in abused babies [47], association with abuse trauma was not statistically proven, and the role the finding plays in the diagnose of abusive head trauma has to be carefully considered on the singular case.

The data from radiological or pathological investigation suggest that cervical injuries are mainly seen in infants while children older than 1 year of age frequently presented with spinal lesions at a lower level, a view that probably indicates that the same mechanism of trauma can lead to different spinal changes according to the age of the victim.

In conclusion, spinal cord investigation is of great interest in forensic assessment of head trauma from abuse with enormous impact on the approach to child autopsies. According to the evidence shown here, forensic pathologists should routinely include the examination of the spinal cord in a systematic approach to AHT cases. Finding the typical intracranial changes of the triad together with spinal lesions will support the diagnosis of abusive head trauma with a higher level of certainty. On the contrary, overlooking the spinal changes could lead to harmful misinterpretation since the triad is nowadays considered not sufficient to support the diagnosis of abuse. Indeed, the view that spinal subdural blood collection is highly suggestive of abuse is in agreement with the clinical practice guidelines on shaken baby syndrome produced by the French Haute Autorité de Santé which underlines the importance of spinal SDH in differentiating abusive from accidental aetiology [71]. In the guidelines, spinal cord lesions in association with unifocal intracranial SDH is considered sufficient to support the diagnosis of AHT within infants in whom differential diagnoses have been ruled out.

In the forensic setting, where objectivity is the leading rule to follow, new objective anatomopathological data are of great interest to help the accurate evaluation of the diagnosis and to allow the court to reach the right verdict. The present work underlined the common finding of spinal blood collection at lower level than previously shown. Consequently, further studies need to be performed in order to better investigate the incidence of spinal blood collection at thoracolumbar level and the origin of it in association to abusive trauma, providing more evidence of the importance of spinal cord injuries in the recognition of abuse.
